# Beyond nutrition: hunger and its impact on the health of young Canadians

**DOI:** 10.1007/s00038-015-0673-z

**Published:** 2015-05-01

**Authors:** William Pickett, Valerie Michaelson, Colleen Davison

**Affiliations:** Department of Public Health Sciences, Queen’s University, Carruthers Hall, Kingston, ON K7L 3N6 Canada; School of Religion, Queen’s University, Kingston, ON Canada; KGH Clinical Research Centre, Kingston General Hospital, Kingston, ON Canada

**Keywords:** Adolescent, Child, Epidemiology, Food, Hunger, Social determinants of health

## Abstract

**Objectives:**

In a large Canadian study, we examined: (1) the prevalence of hunger due to an inadequate food supply at home; (2) relations between this hunger and a range of health outcomes, and; (3) contextual explanations for any observed associations.

**Methods:**

A cross-sectional survey was conducted of 25,912 students aged 11–15 years from 436 Canadian schools. Analyses were descriptive and also involved hierarchical logistic regression models.

**Results:**

Hunger was reported by 25 % of participants, with 4 % reporting this experience “often” or “always”. Its prevalence was associated with socio-economic disadvantage and family-related factors, but not with whether or not a student had access to school-based food and nutrition programs. The consistency of hunger’s associations with the health outcomes was remarkable. Relations between hunger and health were partially explained when models controlled for family practices, but not the socio-economic or school measures.

**Conclusions:**

Societal responses to hunger certainly require the provision of food, but may also consider family contexts and basic essential elements of care that children need to thrive.

## Introduction

Consistent access to a healthy food supply is an important determinant of health and a fundamental human right (World Food Programme 2007). In children, food is required to nurture healthy growth and development but is related to the vitality of the whole person, including their emotional and social well-being (World Food Programme 2007). During the adolescent years, the need for intake of sufficient calories and essential nutrients increases (Dwyer 1993) and food insufficiencies can lead to ongoing health problems (Molcho et al. 2007).

Hunger during childhood is related to poor food and nutritional environments in families (Robaina and Martin 2013; Alaimo et al. 2001). It is also prognostic for negative child health outcomes such as obesity (Nackers and Appelhans 2013), poor self-rated health (Niclasen et al. 2013a, b), increased physical symptoms and associated medicine use (Niclasen et al. 2013a, b), poorer emotional health (Melchior et al. 2012), lower health-related quality of life (Casey et al. 2005), and impaired physical function (Casey et al. 2005). Hunger associated with poor nutrition also makes children vulnerable to disease due to infections (World Food Programme 2007). Manifestations of hunger are sometimes overt, but are often invisible, and reflect the powerlessness and vulnerability of those who suffer from its consequences (World Food Programme 2007).

Hunger caused by an inadequate or inconsistent food supply at home is part of a larger societal issue that is variably referred to as “food insecurity”, “food poverty”, or “food insufficiency” (Food and Agriculture Organization of the United Nations 1996). These terms refer to a myriad of situations where consistent physical and economic access to sufficient, safe and nutritious food of a person’s preference cannot be assured (Food and Agriculture Organization of the United Nations 1996). This field of study is, however, challenging in that there are many different disciplines that are interested and there are subtle differences in the way that these concepts are approached. For example, a recent review suggests that in excess of 200 definitions exist for the term “food insecurity” alone (Food and Agricultural Organization of the United Nations 2013), making comparisons across studies and disciplines daunting.

Further, while the origins and health consequences of hunger have been studied extensively in adults (Stuff et al. 2004; Vozoris and Tarasuk 2003), few analogous large-scale studies exist for adolescents, and almost none in first world countries such as Canada. The etiology of hunger is tied to many obvious social factors including poverty (James et al. 1997) and the strength and organization of families and community networks (Fulkerson et al. 2006). Less evidence exists about the consequences of persistent hunger on the health and well-being of young people. The extent to which organized food and nutrition programs impact upon the long-term effects of hunger is also under-studied (Gelli and Daryanani 2013). Collectively, these represent important gaps in knowledge.

Our Canadian research group is involved in the Health Behavior in School-aged Children (HBSC) study. HBSC is a World Health Organization collaborative study of health and health risk behaviors. We performed a national study of hunger in populations of young people. We explored: (1) the prevalence of self-reported hunger and variations in reported hunger among groups of adolescents; (2) relations between hunger and several physical, emotional and social health outcomes, and; (3) whether such relations can be explained in part by socio-economic, family, and school-based contextual influences. Our aim was to provide foundational information to support the development of evidence-based prevention strategies, and we believe that this was achieved.

## Methods

### Study population and procedures

HBSC involves written health surveys conducted in classroom settings, with a focus on the adolescent years (ages 11–15). It is administered every 4 years following a common international protocol (Freeman et al. 2011).

The 2009–2010 (cycle 6) Canadian sample was developed using a multi-stage, clustered strategy. The primary sampling unit was schools. All students within selected classrooms within those schools were approached to participate. The sample was stratified first by province/territory, then: type of school board (public vs. separate), urban–rural geographic status, school population size, and language of instruction (French vs. English). If a school board or school refused participation, a neighboring school board or school with similar characteristics was approached to participate. Standardized population weights were generated to account for oversampling and stratification criteria. Children from private schools, home schools, First Nations or Inuit reserves, street youth, incarcerated youth, and youth not providing informed consent (explicit or implicit, as per school board customs) were excluded. Response rates were 11/13 (84.6 %) at the province/territorial level (New Brunswick and Prince Edward Island were excluded), 436/765 (57.0 %) at the level of schools (404 of whom answered an administrators’ questionnaire), and 26,078/33,868 (77.0 %) at the student level, with a weighted sample of 25,912 providing responses to a question on hunger.

The study protocol was approved by the Queen’s University General Research Ethics Board. Written parental consent or implied (passive) consent was obtained, according to local school board customs.

### Measures

#### Hunger

Students’ perceptions of hunger were measured: “Some young people go to school or to bed hungry because there is not enough food at home. How often does this happen to you?” with response options of never, sometimes, often, or always. This item was piloted in six HBSC countries (Canada, Macedonia, Norway, Poland, Scotland, Wales) in the year 2000. Findings indicated that young people were interpreting the words of the question as a measure of social deprivation (Griebler et al. 2009). It has been used as an indicator of child hunger, and as well as a proxy indicator of socio-economic status and food availability. With respect to concurrent validity, reports from this hunger measure have been related to the many adverse health behaviors and outcomes (Riches 1997; Molcho et al. 2007; Mullen et al. 2002). It is not, however, possible to relate individual child reports of hunger to other “gold standard” measures.

#### Individual health outcomes

*Adiposity* Youth self-reported weight and height in metric or imperial units and body mass index (BMI) was calculated. To account for growth and maturation, children’s BMI values were converted to age-and sex-specific levels and categorized into normal weight, overweight, or obese using standard international cut-points (Cole 1979).

*Physical inactivity* Moderate to vigorous physical activity was measured by taking an average of the responses to: “Over the past 7 days, on how many days were you physically active for a total of at least 60 min per day?” and “Over a typical or usual week, on how many days are you physically active for a total of at least 60 min per day?” (Prochaska et al. 2001). Physical inactivity was defined as a score of <4 days per week, aligned with the Canadian child physical activity guidelines (Nichol et al. 2009).

*Frequent physical fighting* Reports of two or more physical fights in the past 12 months were used to identify more young people who engaged in violence more frequently. Frequent physical fighting is a validated construct with extensive use in adolescent health surveys (Pickett et al. 2013; Brener et al. 1999).

*Engagement in bullying* and being a *victim of bullying* were assessed using items adapted from Olweus, and defined as perpetrating or being victimized by bullying while at school regularly (2 or 3 times per month to several times per week), following existing precedents (Kyriakides et al. 2006).

*Frequency of talking back to teachers* (4–6 vs. 1–3 on a 6-point scale where 1 was definitely not like me and 6 were definitely like me) was used as a measure of social delinquency.

#### Composite health outcomes

*Psychosomatic symptoms* Youth reported the frequency (5-point Likert-like scale ranging from rarely or never to almost every day) of the following psychosomatic symptoms: headache, stomach ache, backache, feeling low (depressed), irritability or bad temper, nervousness, difficulty in getting sleep, dizziness. These were combined into a composite scale with strong psychometric properties (Hetland et al. 2002). We later divided scores into categories, with the top category representing frequent (on average “weekly” or “daily”) reporting of symptoms.

*Emotional health scales* Four existing composite scales were used to describe negative and then positive aspects of emotional health that involved different internalizing and externalizing behaviors (Freeman et al. 2011). These were conceptualized as follows: internalizing-negative (emotional problems); externalizing-negative (behavioral problems); internalizing-positive (emotional well-being), and externalizing-positive (pro-social behaviors).

#### Demographic factors

Variables considered as descriptive covariates included: gender (boys vs. girls); school grade (6–8 vs. 9–10); immigration status (born in Canada, immigrated >5 years ago, immigrated within 1–5 years); family structure in the primary home (mother and father, mother and step-father, father and step-mother, mother only, father only, other); (all level 1), and geographic size of the residential community [rural (<1000 persons); small (1000–19,999 persons); medium (20,000–99,999 person); and large (≥100,000 persons) (level 2)].

#### Family and school factors

*Socio*-*economic status (level 1)* Family affluence (FAS; a validated measure of socio-economic status) (Currie et al. 2008) was measured by assessing participants’ answers to four items describing the material conditions of their household (respondents’ own household bedrooms, family holidays, family vehicle ownership, family computer ownership). Responses to the items are summed on a nine-point scale with set cut-points for low (0–3), medium (4–5) and high (6–9) affluence.

*Family characteristics (level 1)* Each participant was asked “on average, how many times per week does your family sit down at the table together for dinner/supper?” (response options: zero through seven times) (Elgar et al. 2013). They were also asked “how often do you usually have breakfast (more than a glass of milk or fruit juice) on weekdays?” (response options: never through 5 days) (Tarasuk and Vogt 2009). Participants were also asked about communication in the home, i.e., “How easy is it for you to talk to the following persons (categories included mother, father) about things that really bother you? “(5 response options: “very easy”, “easy”, “difficult”, “very difficult”, “don’t have or see this person”)” (Elgar et al. 2013).

*School food and nutrition programs (level 2)* The HBSC administrator’s questionnaire contained items describing food and nutrition. Three internally consistent scales (Cronbach’s alpha ranged from 0.77 to 0.91) described availability of healthy food choices available at school. Additional school items asked about access to nutritious food regardless of ability to pay; literacy programs related to healthy eating, breakfast and lunch programs, and specific educational opportunities aimed at nutrition (cooking classes, gardening classes, and field trips to grocery stores, farms or farmers markets).

### Statistical analysis

Data analyses were conducted with SAS 9.3 (SAS Institute, Cary, NC, 2010). Descriptive analyses were used to characterize the prevalence of hunger as perceived by students in the overall population, and then within-population subgroups. We also described socio-economic contexts, family characteristics and practices, and the presence of school food and nutrition programs in participating schools and then their relations with hunger.

We used a hierarchical approach to our modeling. The first step was the development of empty models for each of the 11 health outcomes. These partitioned the variance in each of the health outcomes attributable to level 1 (students) nested within level 2 (school) factors.

Next, bivariate logistic regression analyses were then conducted to model each of the 11 health outcomes described above (yes vs. no) as dependent variables, with reports of hunger as the independent variable. These models too accounted for the nested and clustered nature of the sampling scheme using the SAS PROC GLIMMIX procedure and by specifying the schools as random effects. We specified fixed betas but random intercepts after testing the fit of models that were based upon different assumptions. Standardized weights were also applied to account for variations in sampling between provinces and territories.

Next, a series of three separate adjusted logistic regression models that employed the same hierarchical approach were developed to explore the idea that relations between hunger and health could be explained by various factors: (1) family socio-economic conditions (Model 1; with family affluence treated as a level 1 variable); (2) family characteristics and practices (Model 2; with these treated as level 1 variables), and, (3) school food and nutrition programs (Model 3; with these treated as level 2 variables). For ease of interpretation, we adjusted for a standard set of Level 1 confounders in each of the three models (age, grade level, immigration status, and family structure of the primary home), with the latter two models also adjusted for family affluence. Model 1 and Model 2 therefore included only level 1 predictors but included school as a random effect to adjust for clustering. Model 3 included both level 1 and level 2 factors and school as a random effect. When interpreting the findings of the three models, substantial changes (≥10 %) in estimates towards the null for adjusted versus bivariate models were interpreted as evidence in support of the three separate explanations for the occurrence of hunger.

## Results

Overall, a weighted sample of 25,912 young people was included. Approximately 25 % of this sample reported going to school or bed hungry because there was not enough food at home at least “sometimes”, with 3.8 % indicating that this occurred “often” or “always” (Table 1). No substantial variations in these proportions were observed by gender, grade level, immigration status, or community size.

**Table 1:** Hunger reported by young people in Canada: Canadian HBSC Study, 2010

Sub-group	Sample (*n*)	Percent (%) reporting going to school or bed hungry because there is not enough food at home			
		Never	Sometimes	Often	Always
Sample	25,912	74.9	21.4	2.8	1.0
By gender					
Boys	12,708	74.1	22.0	2.8	1.1
Girls	13,198	75.6	20.8	2.7	0.9
By grade level					
6–8	15,523	73.6	22.8	2.7	0.9
9–10	10.388	76.8	19.2	2.8	1.2
By immigration status					
Born in Canada	13,951	75.9	20.5	2.6	0.9
Immigrant: recent	1199	72.7	22.9	2.8	1.6
Immigrant: not recent	6.097	72.0	23.9	3.0	1.1
By family structure of primary home					
Both parents	16,890	77.0	19.9	2.2	0.9
Mother and step-father	1961	72.1	23.7	3.2	1.0
Father and step-mother	506	77.3	19.4	3.0	0.9
Mother only	3805	70.9	24.1	3.9	1.1
Father only	814	65.8	27.6	4.8	1.7
Other	1177	69.0	25.7	3.8	1.4
By geographic center size					
Rural or remote	977	77.4	19.6	1.9	1.1
Small	10,712	75.4	21.3	2.5	0.9
Medium	5688	75.2	21.0	2.8	1.0
Large	8538	74.4	21.4	3.0	1.2

**Fig. 1:**
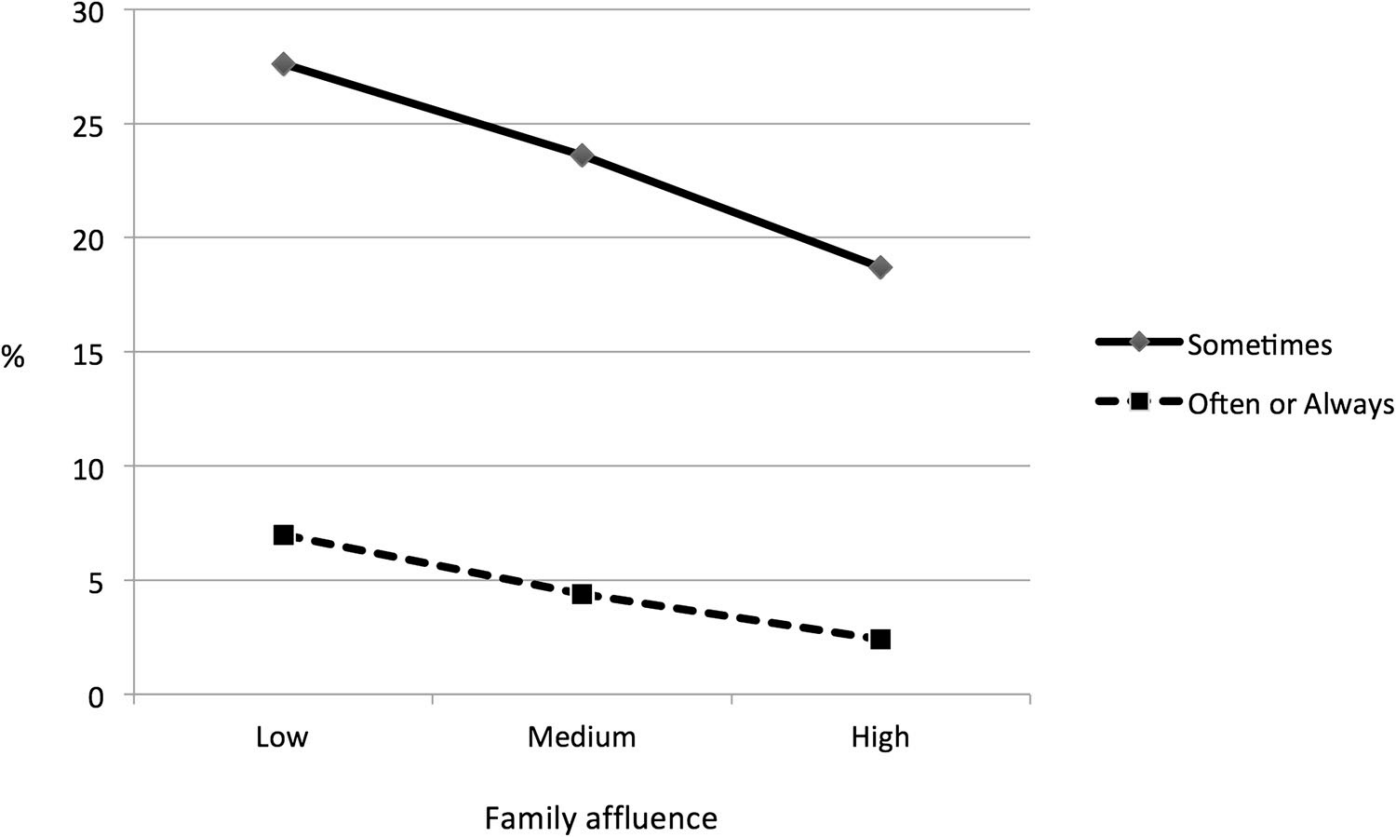
Percentage of young people reporting hunger by level of family affluence: Canadian HBSC Study, 2010

**Fig. 2:**
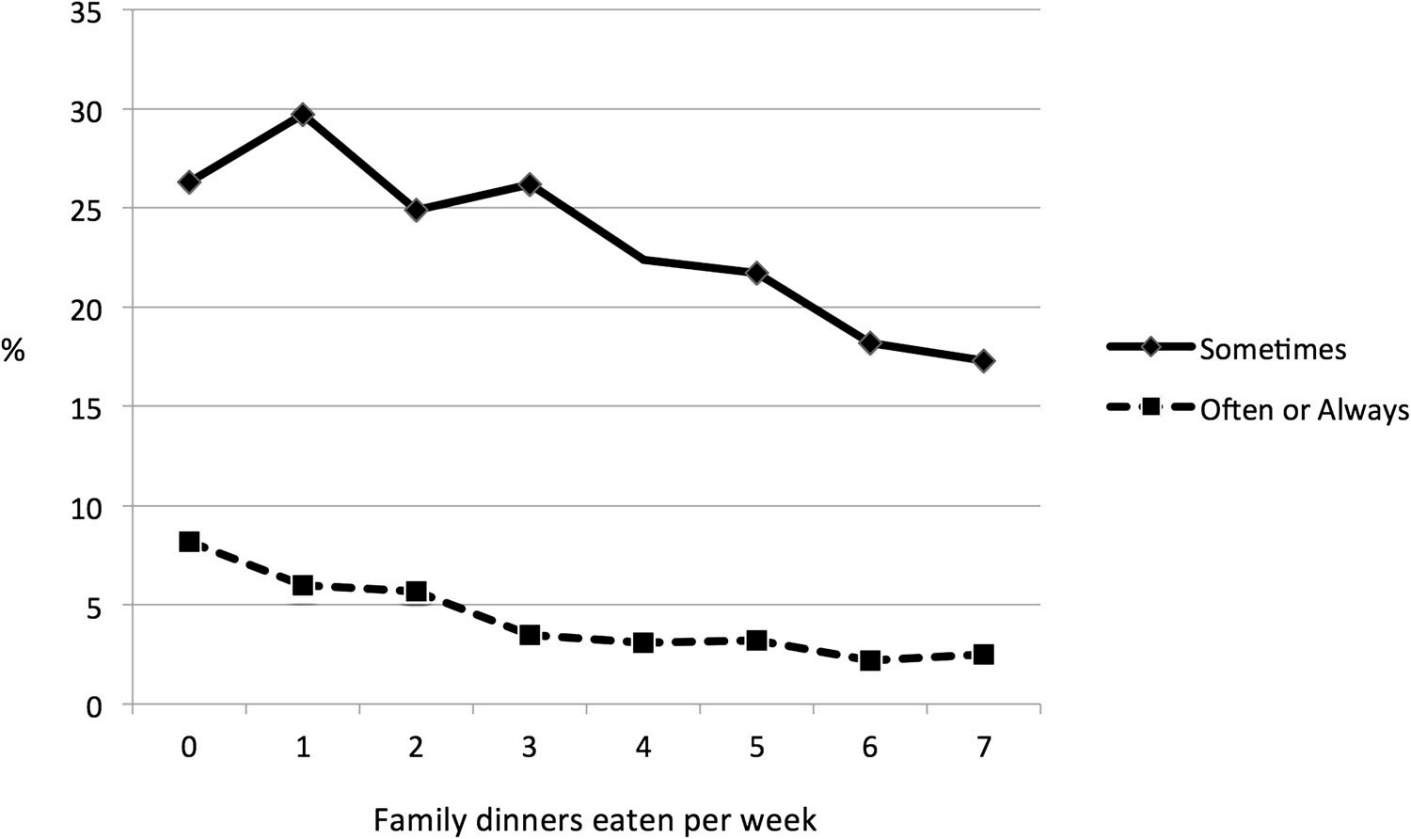
Percentage of young people reporting hunger by number of family dinners eaten together per week at home: Canadian HBSC Study, 2010

Hunger related to a number of socio-economic and family factors, and we present some illustrative examples here. More frequent levels of hunger were reported when respondents came from single parent and “other” versus two parent family structures (Table 1). The prevalence of hunger declined with higher reported levels of family affluence (Fig. 1). Strong and consistent declines in hunger were also observed with increased participation in family meals (Fig. 2). Similar associations were evident between improved levels of family communication and reduced experiences of hunger, and increased breakfast eating and reduced hunger.

**Fig. 3:**
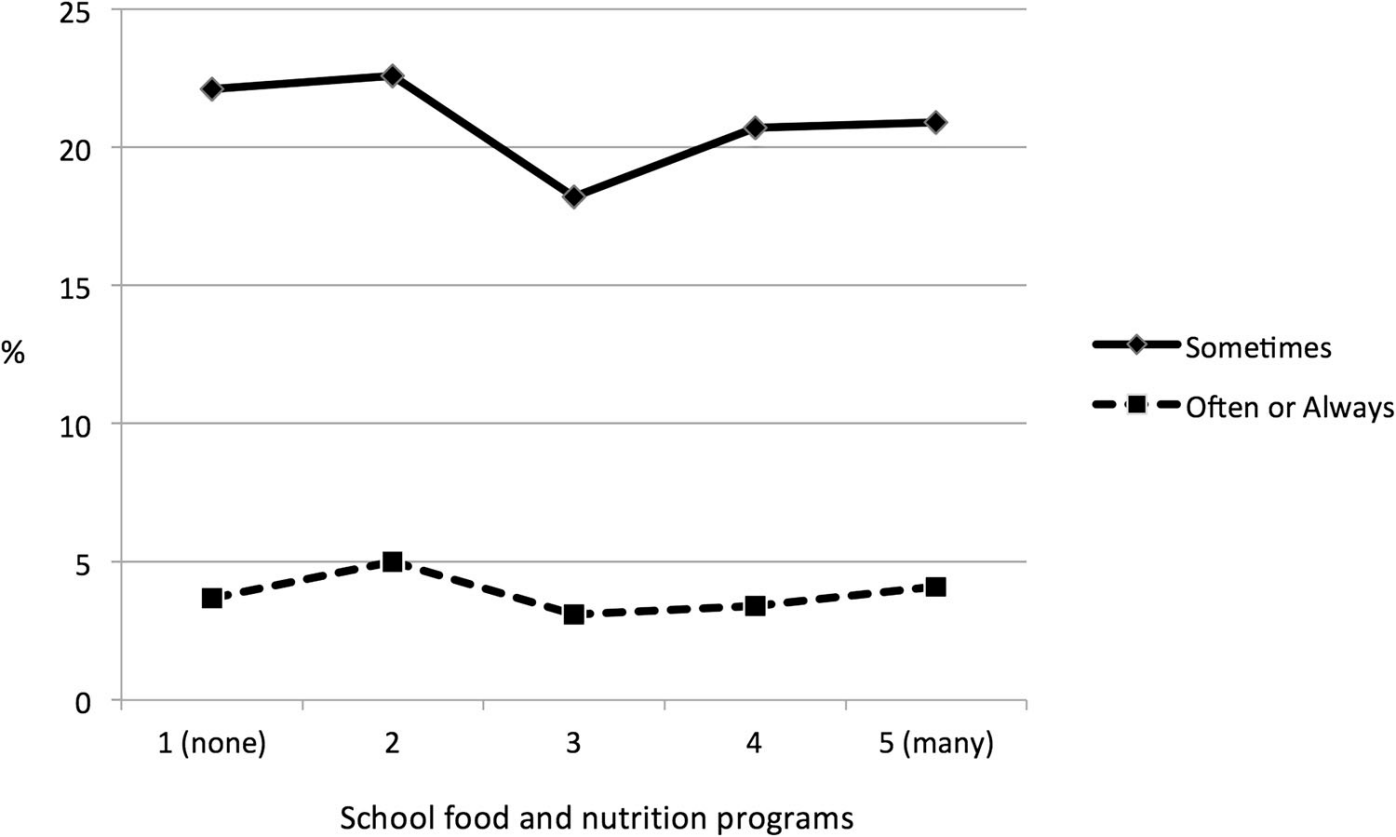
Percentage of young people reporting hunger by reported levels of school food and nutrition programs in school cafeterias: Canadian HBSC Study, 2010

Substantial proportions of Canadian schools are involved in formal efforts to address hunger, food and the nutritional needs of children (Table 2). Formal school programs were aimed at nutritional education, healthy eating, as well as the provision of food at a reasonable cost. However, reported levels of “going to school or bed hungry” were not associated with these school programs in a consistent or strong manner (Fig. 3).

**Table 2:** Food environments reported by school administrators in Canada: Canadian HBSC Study, 2010

Variable	% Yes
Cafeteria food and nutrition programs (*n* = 396 responding schools)	
Healthy food choices at a reasonable or subsidized price	46.5
Healthy eating promotional materials	41.9
Daily healthy eating specials	38.6
Healthy eating program (e.g., eat smart or independent program)	34.3
Other initiative to promote healthy eating	6.6
Snack bar food and nutrition programs (*n* = 396 responding schools)	
Healthy food choices at a reasonable or subsidized price	20.5
Healthy eating promotional materials	13.4
Daily healthy eating specials	6.8
Healthy eating program (e.g., eat smart or independent program)	5.6
Other initiative to promote healthy eating	2.5
Vending machine food and nutrition programs (*n* = 396 responding schools)	
Healthy food choices at a reasonable or subsidized price	23.2
Healthy eating promotional materials	6.1
Daily healthy eating specials	2.0
Healthy eating program (e.g., eat smart or independent program)	2.5
Other initiative to promote healthy eating	0.8
Other food and nutrition initiatives (number of responding schools varies)	
All students, regardless of ability to pay, have access to fruits and vegetables (*n* = 396)	
At least sometimes during year	67.3
Entire year	39.9
Occasional or seasonal	27.3
Media literacy related to healthy eating (*n* = 404)	67.3
Healthy food choices during lunch program (*n* = 391)	66.8
Healthy food choices during breakfast program (*n* = 387)	57.9
Cooking classes (*n* = 404)	58.9
Field trips to local grocery stores (*n* = 404)	35.6
Field trips to farms or farmers markets (*n* = 404)	26.5
Gardening classes (growing produce) (*n* = 404)	15.3

Relations between the self-reported level of hunger and health outcomes are summarized below and in Table 3. The percentage of variance that was observed at the school level varied from 0.9 to 10.3 %, justifying the use of a multi-level or hierarchical modeling approach. Results indicated by the bivariate models were remarkably consistent. Each showed statistically significant increases in risk for all negative individual and composite health outcomes studied, and similar decreases in risk for the two positive individual health outcomes. Adjusted Model 1 shows that relations between hunger and health remained basically unchanged when we controlled for our level 1 measure of self-reported family affluence. Adjusted Model 2 indicated consistent changes in the odds ratio estimates towards the null for all 11 outcomes once the level 1 family variables were controlled for. These included measures of frequency of engagement in family dinners, breakfasts eaten by young people during the school week, and ease of communication with mothers and fathers. Adjusted Model 3 shows that the odds ratio estimates remained relatively unchanged from the bivariate estimates following adjustment for level 2 indicators of school food and nutrition programs. The latter included summary scales describing food availability at school, and individual measures describing access to food at school regardless of ability to pay, breakfast and lunch programs, and classes that focused on diet and nutrition.

**Table 3:** Relationships between hunger and individual and composite physical, emotional and social health outcomes: Canadian HBSC Study, 2010

Health outcome: level of food insecurity	% with Health outcome	OR (95 % CI)^d^			
		Bivariate Models	Adjusted Model 1: family socio-economic conditions^a^	Adjusted Model 2: family characteristics and practices^b^	Adjusted Model 3: school food and nutrition programs^c^
Individual health outcomes					
Overweight/obese					
Never	20.0	1.00	1.00	1.00	1.00
Sometimes	24.4	1.28 (1.18–1.39)	1.26 (1.16–1.37)	1.25 (1.14–1.36)	1.25 (1.14–1.37)
Often or always	24.9	1.33 (1.11–1.58)	1.23 (1.01–1.48)	1.14 (0.94–1.39)	1.28 (1.05–1.56)
Physically inactive					
Never	33.4	1.00	1.00	1.00	1.00
Sometimes	39.7	1.28 (1.20–1.37)	1.24 (1.16–1.33)	1.15 (1.06–1.23)	1.23 (1.14–1.32)
Often or always	44.8	1.53 (1.34–1.76)	1.44 (1.24–1.67)	1.31 (1.12–1.53)	1.41 (1.20–1.60)
Frequent physical fighting					
Never	17.9	1.00	1.00	1.00	1.00
Sometimes	22.7	1.36 (1.26–1.46)	1.32 (1.21–1.43)	1.18 (1.08–1.29)	1.30 (1.19–1.42)
Often or always	26.5	1.66 (1.42–1.94)	1.76 (1.48–2.09)	1.42 (1.19–1.70)	1.81 (1.51–2.16)
Frequent bullying					
Never	14.7	1.00	1.00	1.00	1.00
Sometimes	18.9	1.33 (1.23–1.45)	1.31 (1.20–1.42)	1.15 (1.05–1.26)	1.31 (1.20–1.44)
Often or always	29.0	2.33 (2.00–2.70)	2.31 (1.96–2.72)	1.76 (1.48–2.09)	2.31 (1.95–2.74)
Frequent victimization by bullying					
Never	26.0	1.00	1.00	1.00	1.00
Sometimes	38.5	1.80 (1.68–1.92)	1.76 (1.66–1.89)	1.62 (1.51–1.75)	1.81 (1.69–1.95)
Often or always	48.0	2.74 (2.39–3.13)	2.53 (2.19–2.93)	2.06 (1.77–2.40)	2.61 (2.24–3.04)
Talk back to teachers					
Never	17.8	1.00	1.00	1.00	1.00
Sometimes	23.4	1.34 (1.24–1.46)	1.33 (1.22–1.45)	1.25 (1.14–1.37)	1.31 (1.20–1.44)
Often or always	30.6	1.93 (1.63–2.27)	1.73 (1.45–2.06)	1.50 (1.25–1.81)	1.78 (1.48–2.15)
Composite health outcomes					
Frequent psychosomatic symptoms					
Never	24.1	1.00	1.00	1.00	1.00
Sometimes	35.4	1.78 (1.66–1.90)	1.84 (1.71–1.99)	1.56 (1.44–1.69)	1.88 (1.74–2.04)
Often or always	55.1	3.85 (3.35–4.43)	4.27 (3.65–4.98)	3.24 (2.74–3.84)	4.42 (3.75–5.21)
Internalizing problems					
Never	29.0	1.00	1.00	1.00	1.00
Sometimes	45.7	2.09 (1.97–2.23)	2.15 (2.01–2.30)	1.89 (1.76–2.03)	2.12 (1.97–2.27)
Often or always	60.4	3.78 (3.30–4.33)	3.82 (3.29–4.44)	2.96 (2.52–3.48)	3.78 (3.23–4.42)
Externalizing problems					
Never	33.8	1.00	1.00	1.00	1.00
Sometimes	39.9	1.28 (1.19–1.36)	1.24 (1.15–1.33)	1.11 (1.03–1.19)	1.21 (1.12–1.30)
Often or always	52.4	2.02 (1.75–2.33)	2.00 (1.72–2.32)	1.59 (1.36–1.86)	2.05 (1.75–2.40)
Emotional well-being					
Never	40.0	1.00	1.00	1.00	1.00
Sometimes	28.5	0.56 (0.53–0.60)	0.56 (0.52–0.61)	0.67 (0.62–0.73)	0.58 (0.53–0.62)
Often or always	22.5	0.42 (0.36–0.49)	0.45 (0.38–0.54)	0.55 (0.45–0.67)	0.44 (0.36–0.53)
Pro-social behavior					
Never	32.6	1.00	1.00	1.00	1.00
Sometimes	28.2	0.81 (0.75–0.86)	0.82 (0.77–0.88)	0.89 (0.83–0.96)	0.84 (0.78–0.90)
Often or always	29.0	0.85 (0.73–0.99)	0.83 (0.71–0.98)	0.88 (0.74–1.04)	0.85 (0.72–1.01)

^a^Adjusted Model 1. Adjusted for level 1 variables of age, gender, immigration status, family structure, and family affluence

^b^Adjusted Model 2. Adjusted for level 1 variables of age, gender, immigration status, family structure, family affluence, communication with father, communication with mother, family dinners, frequency of breakfast consumption

^c^Adjusted Model 3. Adjusted for level 1 variables of age, gender, immigration status, family structure, family affluence, and level 2 variables of school breakfast and lunch programs, availability of food at cost, cooking classes, gardening classes, school cafeteria programs, school snack bar programs

^d^All models also include school as a random effect to account for clustering

## Discussion

This national study demonstrated that hunger was common among young Canadians. It confirmed the presence of relations between hunger and poverty, but perhaps less predictably, showed that hunger is also related to a number of common family characteristics including their structure, communication patterns, and meal practices, irrespective of socio-economic status. We also confirmed the existence of strong relations between going to school or bed hungry and a large number of negative health outcomes. And finally, we showed that while these latter relations were in part accounted for by family characteristics, there was no evidence of similar mediation effects by our measures of socio-economic status or our assessment of school-based food and nutrition programs.

Basic information on the prevalence of hunger is informative. In a developed country such as Canada, it is remarkable that up to 25 % of young people report going to school or bed hungry due to a lack of food in the home at least occasionally, with 4 % reporting this often or always. Even considering the opportunity for misclassification, these estimates are sobering. By extrapolation and using population counts from the 2011 Census of Population (Statistics Canada 2014), the 3.8 % figure translates into a total of 73,000 Canadian children aged 11–15 years reporting going to school or bed hungry as indicated by our measure (54,000 “often”, 19,000 “always”). Reported prevalence levels are consistent with historical reports for child populations (Tarasuk and Vogt 2009), and they point to a quiet public health problem in a wealthy country that shows that hunger is not just experienced in economically disadvantaged nations.

We also confirmed a number of gradients consistent with the idea that hunger has social origins. We and others (Molcho et al. 2007) have shown that hunger due to having insufficient food at home occurs not only in disadvantaged families, but in affluent families as well. In addition, our analyses did not find expected relations between school-based food and nutrition programs and going to school or bed hungry. While this was somewhat expected because the wording of the question specified hunger in non-school hours, it does point to possible limitations of school programs for experiences of overall hunger, and may suggest a more distal role of schools in this etiological pathway. This is not to say that programs that provide food to children are not of value. Rather, it suggests that hunger is a more ubiquitous problem, and while concentrated within impoverished families and a spectrum of schools who serve socio-economically disadvantaged populations, it is not unique to them. Hunger crosses cultures and populations, and feeding the hungry in all parts of society should always remain a priority (US Department of Agriculture 2013; Melbye et al. 2013).

Going to school or bed hungry was also remarkably consistent in its associations with negative health outcomes. It is telling that these relations persisted when we controlled for our available measure of socio-economic status. This suggests that poverty alone is less likely to explain why hunger results in various states of impaired health. Other social explanations are warranted. Next, when we controlled for school-based food and nutrition programs, the observed negative health relations persisted. While such programs address an obvious need, they do not eliminate the persistent negative health effects of a home without adequate organization or resources to ensure a consistent and adequate food supply. While addressing child hunger directly with food and nutrition programs is an essential moral and social responsibility, it is only part of the solution to a more complex social problem.

Negative health outcomes related to hunger may be caused not only by lack of food, but by more insidious feelings caused by food insecurity at home. Relations between hunger and the various health effects were attenuated, and perhaps partially mediated, when home-based factors were controlled for in our models. Speculatively, much of the challenge in addressing this hunger problem lies with addressing social factors that originate in the home. The latter may include things such as the stress that comes with having limited or sporadic access to food as well as feelings of a lack of control in life, injustice, questioning of one’s self worth, stress around problems of food access, and associated alienation (Coates et al. 2006).

A more holistic approach has been proposed to address the negative effects of hunger on adolescents, with a focus on the concept of “care” (Longhurst and Tomkins 1995). The UN declaration of the rights of the child echoes this basic holistic need (United Nations 1959). Beyond the right to adequate nutrition, a focus on care recognizes that for optimal development, children need love and understanding as well as “an atmosphere of affection and of moral and material security” (United Nations 1959). The role of care in terms of affection, emotional support, and effective allocation of resources within an atmosphere of stability and security has a direct influence on child nutritional outcomes (Engle et al. 2000; Engle and Lida 1999). Even in situations of poverty where there is household food insecurity and children are exposed to unhealthy physical and social environments, providing enhanced care can improve nutritional outcomes (Longhurst and Tomkins 1995).

In the 1990s, care was introduced as a fundamental component of nutritional well-being in young children (Engle et al. 2000; United Nations Children’s Fund 1990). Care has more recently been understood as the behaviors and practices by caregivers to provide the food, health care, stimulation and emotional support that are needed for optimal child health and development (Engle et al. 2000). Ensuring adequate care also involves provision of the time, attention and support required to meet a range of needs in the developing child (physical, mental, emotional and social) (Longhurst and Tomkins 1995). This suggests the need for an integrative approach to the mediation of the consistent negative effects of hunger in children. A holistic approach would certainly include the provision of food, but would also look at the family context and the basic essential elements of care that children require.

If the problem of food insecurity is in the home, innovative solutions to help families are needed there, and not just in community settings. Providing food is one simple—though essential—solution. The root of the issue—the essential human issue—is much more complex. It is connected to our value as human beings and to equity and well-being; not just in filling empty stomachs. In a developed country of such wealth and relative peace, the existence of hunger among children in Canada is a moral failing on the part of society. For the good of all, addressing all aspects of this complex issue needs to become an essential priority, not only by government, but by community leaders, educators, and all capable adults.

Strengths of our study include the size of the study population and its national scope. The analysis was novel and addressed several practical questions about hunger, its distribution within adolescent society, its possible health consequences, and potential explanations. Limitations include the cross-sectional HBSC design, which is not as ideal as longitudinal designs for causal inference. Our use of a single item to measure a very complex construct will result in some misclassification. It is certainly possible that food is available in homes, and children report going to school or bed hungry because they do not like the selection of food available, or do not wish to prepare it for consumption. A strength of this child measure is that it is based upon a simple question that is asked directly to children. This might be less likely to be subject to parental reporting biases. We also had no information on adult factors that could contribute to family dysfunction (e.g., violence, mental health) and then hunger. Finally, the HBSC sampling strategy excluded some adolescent groups that may be at higher risk for hunger (e.g., youth on reserves and street youth), which may impact upon the external validity of our findings.

### Conclusion

In this national study of adolescent Canadians, we document the prevalence of going to school or bed hungry due to having insufficient food at home. This type of hunger was related to a number of negative emotional, physical and social health outcomes. Such relations are modified by family practices, but less so by socio-economic factors or school-based food and nutrition programs. Future research, both quantitative and qualitative, is required to understand fully the social circumstances that result in adolescent hunger. Our findings also point to a need for a focus on practical family measures to truly ameliorate the root causes of this problem and its immediate consequences in terms of child health. While food programs are one important component of addressing hunger in Canadian children, we suggest that a more integrative approach with a focus on care is needed.
